# The vision for public health dietitians’ skill improvement over the next 10 years in Japan: A qualitative study

**DOI:** 10.1016/j.puhip.2023.100392

**Published:** 2023-05-26

**Authors:** Tatsuya Koyama, Yusuke Arai, Ayaka Iida, Sumie Isobe, Okamoto Rie, Izumi Shibuya, Kazumi Tanaka, Ayumi Morooka, Katsushi Yoshita

**Affiliations:** a Aomori University of Health and Welfare, Japan; b Chiba Prefectural University of Health Science, Japan; c Kanagawa University of Human Services, Japan; d Niigata Prefecture Government, Japan; e Kanazawa University, Japan; f Aichi Prefectural Government, Japan; g Hyogo Prefectural Government, Japan; h Osaka Metropolitan University, Japan

**Keywords:** Public health dietitian, Goal, Skill improvement, Position, Human resource development

## Abstract

**Objectives:**

To obtain the basis for developing a new human resource development program. We examined the association between their position type and their vision for skill improvement in the profession in the next 10 years.

**Study design:**

This was a qualitative study.

**Methods:**

In 2021, we conducted an exhaustive survey of Japanese public health dietitians working in Japanese local governments. Using qualitative content analysis, we analyzed the participants’ descriptions of how the profession could improve their skills over the next 10 years.

**Results:**

Regardless of the participants’ organization of employment or their target position type, seven common categories were extracted; [goals], [health promotion activities], [organizational activities], [evaluation from others], [cooperation], [skills to be acquired], and [means for improving skills]. Depending on the organization type, 35–40 subcategories were extracted from those who wanted to be staff, 35–38 subcategories from those who wanted to be supervisors, and 20–37 subcategories from those who wanted to be managers. Different subcategories were extracted to describe the difference between specialists and generalists in [goals]. Participants described challenges with [evaluation from others] and [collaboration], regardless of the target position type or [goals].

**Conclusion:**

The vision for Japanese public health dietitians’ skill improvement to achieve in the next 10 years describes challenges with business evaluation and collaborative work. However, participants differed across what skills they wanted to improve based on the direction of their careers. To offer public health dietitians learning content that connects with their desired direction, a new human resource development program needs to be considered.

## 1 Introduction

Public health dietitians who work for prefectures, cities and special wards that have public health centers, municipalities mainly specialize in developing systems related to nutrition and dietary habits, clarifying health and nutrition issues, promoting measures to prevent the onset and aggravation of lifestyle-related diseases based on the PDCA cycle and improving the social environment [1]. Local governments facilitate a staged growth process of public health dietitians according to their years of service and their efforts to improve their qualifications [[2], [3], [4], [5], [6], [7]].

The public health department of the Japan Dietetic Association conducted focus groups to examine the competencies of public health dietitians, subsequently summarizing them according to years of experience [8]. Furthermore, when developing a human resource development program that takes into consideration the circumstances surrounding public health dietitians, it is important to consider not only the competencies necessary for public health dietitians but also the vision for skill improvement that public health dietitians should aim to achieve in 10 years. The number of municipal dietitians is generally small and often a one-person position in a particular region or organization. Thus, therefore, individual competencies and skills can have a strong influence on the quality of public nutrition activities in a region or organization for years or decades. Municipal dietitians are expected to have sufficient knowledge and skills, depending on their years of service and position, so that they can best respond to the roles expected of them by the community or organization and to the various challenges that arise and change over time. Few systematic survey about the vision of public health dietitians has been conducted on a national scale in Japan to date. Therefore, to contribute to the development of effective training programs for public health dietitians, we collected data on the actual conditions and individual needs of public health dietitians to guide human resource development. In this study, we examined the issues that arise from visions for skill improvement that public health dietitians want to achieve over the next 10 years.

Depending on the type of organization that public health dietitians work for, their roles and future career trajectories differ across Japan [1]. Depending on their trajectory, their ideas about the future vision for Japanese public health dietitians' skill improvement over the next 10 years may differ. This study aimed to clarify the similarities and differences in descriptions of these visions for each organization types’ public health dietitians.

## 2 Methods

### 2.1 Participants

We conducted a cross-sectional survey among dietitians working in prefectures, special wards and cities with public health centers, and municipalities in Japan. The inclusion criteria for the study participants were: (1) those who responded as public health dietitians in a survey by the Ministry of Health, Labour and Welfare; and (2) those with a full-time or part-time (at least four days a week and at least 6 h a day) employment status. However, those currently working full-time in fields such as childcare, welfare for older adults, boards of education, and medical care were excluded.

Request letters were sent in January 2021 to dietitians at supervise bureaus who met the inclusion criteria. In the request letters, we specified the purpose of the survey and the URL and QR code linked to the online questionnaire. The web-survey was conducted from January 29, 2021, to March 2, 2021 (Survey Research Center Co., Ltd.). A request for cooperation was presented at the beginning of the questionnaire form. The request clearly stated the purpose of the survey and outlined that all responses and participation would be anonymous, cooperation in the survey was voluntary, responses would be regarded as consent, and that there would be no disadvantage for non-responses. Additionally, since there was a possibility that personally identifiable information may be transmitted to us when returning responses, we asked the commissioning company to create screens related to the questionnaire responses and receive all replies. This meant that the researchers would only receive simply entered databases obtained by the consignment company, and no identifiable respondent information, such as email addresses. All information was destroyed after the survey was completed and secondary use of the data for other purposes was prohibited.

Public awareness and cooperation regarding the survey was obtained from the public health department of the Japan Dietetic Association and the Japan association of public health center registered dietitians.

### 2.2 Survey item

The survey included items regarding age and length of service for each main type of work. Additionally, dietitians working in prefectures, and cities and special wards with public health centers were asked about the length of duties mainly responsible for community health promotion and improving nutrition and dietary habits at the health department (the headquarter), public health centers, and health centers. We asked dietitians working in municipalities about the length of their main duties, such as community health promotion and improving nutrition and dietary habits at health centers.

We asked participants about their desired position type with the following options: staff member (current position continuation), supervisors (assistant chiefs, central office chiefs, health center section chiefs) and managers (assistant section chiefs, chiefs and above).

It takes ten years or more to gain enough real-world work experience to be an established public health dietitian. It also takes significant time to better understand their talents, motivations and values [9]. Due to this, we asked the participants to freely describe their vision for the skill improvement that they wanted to achieve in 10 years.

### 2.3 Statistical analysis

This study utilized reflexive thematic analysis. Reflexive thematic analysis consists of the following six recursive stages: (1) familiarization with the data, (2) initial coding, (3) generation of the first theme, (4) review and development of the theme, (5) refinement and naming of the theme, and (6) writing. Specifically, the first author read the free-text descriptions for the data (stage 1) and then generated the codes (stage 2). The codes were then aggregated into subcategories of potential semantic patterns, which were further aggregated into categories (stage 3). The relationships among the categories and the categories' conformity to the presupposed story were then examined (stage 4), and the scope and content of the themes were refined (stage 5).

The survey completed by participants included free text, with the text getting divided into sentences that could have several meanings in the analysis, which were then converted into data. The unit of analysis for this study was text and was structured by deleting unnecessary words, generalizing and consolidating the writing style and supplementing explanations. Subcategories were created by collectively classifying and naming them. A subcategory is the smallest unit of data that can be analyzed, and has the potential to be grouped into a single category. A category is the largest unit that can be analyzed, with the subcategories grouped together and given appropriate names. Categories and subcategories were added to the above procedure, and the frequencies were counted.

## 3 Results

### 3.1 Characteristics

Table 1 describes charcteristics of the participants. Responses were obtained from 451 public health dietitians working in prefectures, 323 working in cities and special wards with public health centers, and 1031 in municipalities. The exact number of Japanese public health dietitians employed in community health promotion is unknown, but according to the 2019 Regional Health and Health Promotion Project Report, 703 public health dietitians worked in prefectures, 913 worked in cities and special wards with public health centers, and 2355 worked in municipalities [11]. Using this as a parameter, the response rate of this survey was estimated to be 64.2% for prefectures, 35.3% for cities and special wards with public health centers, and 43.8% for municipalities.

**Table 1:** Characteristics of participants.

		Working in prefectures						Working in cities with public health centers and special wards						Working in municipalities					
		Desired position						Desired position						Desired position					
		Staff (n = 214)		Supervisor (n = 183)		Manager (n = 54)		Staff (n = 166)		Supervisor (n = 135)		Manager (n = 22)		Staff (n = 521)		Supervisor (n = 328)		Manager (n = 182)	
		n	%	n	%	n	%	n	%	n	%	n	%	n	(%)	n	(%)	n	(%)
Age	20s	48	(22)	51	(28)	11	(20)	31	(19)	34	(25)	4	(18)	124	(24)	88	(27)	37	(20)
	30s	39	(18)	34	(19)	6	(11)	50	(30)	33	(24)	4	(18)	178	(34)	83	(25)	51	(28)
	40s	57	(27)	48	(26)	15	(28)	44	(27)	36	(27)	4	(18)	144	(28)	111	(34)	60	(33)
	Over 50s	70	(33)	50	(27)	22	(41)	41	(25)	32	(24)	10	(46)	75	(14)	46	(14)	34	(19)
Local health promotion and improvement of nutrition and eating habits at the hygiene administration department (main government office)	No work experience	137	(63)	89	(49)	22	(41)	103	(62)	74	(55)	10	(45)	-	-	-	-	-	-
	<3 years	37	(17)	35	(19)	8	(15)	28	(17)	21	(16)	4	(18)	-	-	-	-	-	-
	3–5 years	30	(14)	34	(19)	9	(17)	12	(7)	16	(12)	3	(14)	-	-	-	-	-	-
	5–10 years	8	(4)	15	(8)	8	(15)	13	(8)	16	(12)	5	(23)	-	-	-	-	-	-
	10–20 years	1	(0)	7	(4)	7	(13)	7	(4)	7	(5)	0	(0)	-	-	-	-	-	-
	20–30 years	1	(0)	2	(1)	0	(0)	2	(1)	1	(1)	0	(0)	-	-	-	-	-	-
	+30 years	0	(0)	1	(1)	0	(0)	1	(1)	0	(0)	0	(0)	-	-	-	-	-	-
Community health promotion and improvement of nutrition and dietary habits at public health centers and health centers	No work experience	5	(2)	5	(3)	1	(2)	4	(2)	8	(6)	1	(5)	68	(13)	44	(13)	16	(9)
	<3 years	45	(21)	40	(22)	8	(15)	45	(27)	31	(23)	5	(23)	165	(32)	75	(23)	41	(23)
	3–5 years	27	(13)	20	(11)	7	(13)	23	(14)	18	(13)	3	(14)	69	(13)	44	(13)	20	(11)
	5–10 years	31	(14)	33	(18)	11	(20)	41	(25)	31	(23)	4	(18)	103	(20)	55	(17)	31	(17)
	10–20 years	61	(29)	56	(31)	15	(28)	35	(21)	31	(23)	3	(14)	80	(15)	60	(18)	43	(24)
	20–30 years	34	(16)	27	(15)	11	(20)	12	(7)	15	(11)	5	(23)	31	(6)	48	(15)	28	(15)
	+30 years	11	(5)	2	(1)	1	(2)	6	(4)	1	(1)	1	(5)	5	(1)	2	(1)	3	(2)

### 3.2 The vision for public health dietitians’ skill improvement in the next 10 years

We extracted 2134 codes and analysis of these codes resulted in the formation of 40 subcategories. The subcategories were further grouped according to similarity, resulting in the formation of seven categories. Below, categories in the text are indicated by [] and subcategories are indicated by ““.

Six categories were extracted from the free descriptions of what participants should be in 10 years and their skill development. [Goals] were formed from the goal descriptions as individuals (specialists and generalists) and as general public health dietitians. [Health promotion activities] was a category that described activities for people in the community over the next 10 years, including specific activities such as “nutrition education”, “food environment”, “community assessment”, “health crisis management, “healthy environment”, and vague descriptions such as “public nutrition activities in general'' and “public health activities in general”. [Organizational activities] was formed from the subcategories of “improvement of work efficiency”, “developing human resources”, and “improvement of working environment”, as they were described as activities to be undertaken in the organization. [Evaluation from others] indicated that the existence, importance, and necessity of public health dietitians were recognised, and that other occupations in the same organization and local residents trusted public health dietitians. In addition, to obtain appropriate evaluations from others, they want to have an opportunity to describe how they would like to appeal to those around them and to explain their achievements. [Collaboration] was described as wanting to cooperate in future activities with others, which could occur within the same organization or in different organizations. [Skills to be acquired] were classified into 13 subcategories by specifically describing what participants wanted to improve within their skillset. [Means for improving skills] described how participants want to improve their skills, such as “participation in workshops”, “self-improvement” and “job rotation” within the organization.

Among the public health dietitians working in prefectures that want to continue their current positions as staff, the following was frequently described: “generalist” as [goals] and “public nutrition activities in general” and “public health activities in general” as [health promotion activities], “human resource development” as [organizational activities], “visualization of achievements” as [evaluation from others], “collaboration with dietitians”, “collaboration with stakeholders” and “collaboration with other occupations” as [collaboration] and “expert knowledge/skill” and “administrative knowledge/skill” as [skills to be acquired] (Table 2). Those who wanted to be in a supervisory position frequently described: “generalist” as the [goals], “nutrition education”, “food environment”, “community assessment”, “health crisis management”, “healthy environment”, “public nutrition activities in general”, and “public health activities in general” as [health promotion activities], “improvement of work efficiency” as [organizational activities]. Those who desired a managerial position frequently described: “specialist”, “generalist”, “establishment and improvement of the status and significance of public health dietitians”, “increasing and securing public health dietitians”, “placement of public health dietitians outside health and hygiene related departments” as [goals] “public nutrition activities in general” and “public health activities in general” as [health promotion activities], and “evaluation within the organization” as [evaluation from others], “collaboration with dietitians” and “collaboration with other occupations” as [collaboration] and “expert knowledge/skill”, “ideas that respond to the times, flexible ideas” as [skill to be acquired].

**Table 2:** The vision and skill improvement that public health dietitians working in prefectures want to achieve in 10 years.

Category	Subcategory	Desired position					
		Staff (n = 214)		Supervisor (n = 183)		Manager (n = 54)	
		n	(%)	n	(%)	n	(%)
Goals	Specialist	4	[2]	8	[4]	3	[6]
	Generalist	11	[5]	19	[10]	13	[24]
	Establishment and improvement of status and significance of public health dieticians	8	[4]	12	[7]	7	[13]
	Increase and secure public health dieticians	7	[3]	12	[7]	5	[9]
	Establishment and expansion of public health dieticians work	3	[1]	5	[3]	1	[2]
	Assignment of public health dietitians to departments other than health and hygiene-related departments	2	[1]	7	[4]	4	[7]
Health promotion activities	Nutrition education	3	[1]	0	(0)	0	(0)
	Food environment	5	[2]	1	[1]	0	(0)
	Community assessment	3	[1]	5	[2]	0	(0)
	Health emergency management	8	[4]	2	[1]	0	(0)
	Healthy environment	1	(0)	1	[1]	0	(0)
	Public nutrition activities in general	11	[5]	5	[3]	2	[4]
	Public health activities in general	14	[7]	6	[3]	3	[6]
Organizational activities	Improvement of work efficiency	3	[1]	3	[2]	0	(0)
	Human resource development	12	[6]	18	[10]	2	[4]
	Creating a comfortable working environment	5	[2]	4	[2]	2	[4]
Evaluation from others	Evaluation from organization	6	[3]	8	[4]	5	[9]
	Evaluation from residents	4	[2]	2	[1]	1	[2]
	Visualization of achievements	11	(5	12	[7]	2	[4]
	Appeal achievements to others	1	(0)	13	[7]	1	[2]
Collaboration	Collaborate with dietitians	14	[7]	12	[7]	4	[7]
	Cooperation with stakeholders	14	[7]	4	[2]	1	[2]
	Cooperation and cooperation with residents	3	[1]	1	[1]	0	(0)
	Collaborate with other occupations	21	[10]	13	[7]	3	[6]
Skills to be acquired	Expert knowledge/skills	13	[6]	8	[4]	3	[6]
	Acquisition of qualification	1	(0)	1	[1]	0	(0)
	Ideas that respond to times and flexible idea	6	[3]	5	[3]	4	[7]
	Ability to show business	6	[3]	2	[1]	0	(0)
	Communication skill	9	[4]	1	[1]	1	[2]
	Presentation skill	3	[1]	0	(0)	0	(0)
	Administrative knowledge/skill	10	[5]	2	[1]	2	[4]
	General knowledge	3	[1]	0	(0)	0	(0)
	Planning and formation of project skill	7	[3]	8	[4]	1	[2]
	Negotiation/coordination skill	4	[2]	1	[1]	0	(0)
	Evaluation analysis ability	3	[1]	3	[2]	1	[2]
	Consensus building ability	1	(0)	0	(0)	0	(0)
	Problem analysis ability	1	(0)	5	[3]	1	[2]
Means for improving skills	Workshop participation	6	[3]	6	[3]	0	(0)
	Self-improvement	3	[1]	0	(0)	0	(0)
	Job rotation	4	[2]	4	[2]	1	[2]

Public health dietitians working in cities and special wards with public health centers who want to continue their current positions as staff described becoming a “specialist” and “generalist” as a [goals], “evaluation within the orgnization” as [evaluation from others], “collaboration with dietitians”, “collaboration with other occupations” as [collaboration], “expert knowledge/skill” and “ideas that respond to the times, flexible idea” as [skill to be acquired] (Table 3). Those who wanted to be in a supervisory position frequently described “human resource development” as [orgnizational activities], “visualization of achievements” as [evaluation from others], and “collaboration with dietitians”, “cooperation with stakeholders‘, “collaboration with other occupations” as [collaboration], “expert knowledge/skill’, “administrative knowledge/skill, and “ideas that respond to the times, flexible ideas as [skills to be acquired] ‘, and “participation in workshops” as [means for improving skills]. Among those who desired a managerial position, “human resource development” and “improvement of working environment” as [organizational activities], and “evaluation from residents” as [evaluation from others], “cooperation with dietitians”, “cooperation with other occupation” as [cooperation], and “participation in workshops” as [means for improving skills] was frequently described.

**Table 3:** The vision and skill improvement that public health dietitians working in cities and special wards with public health centers want to achieve in 10 years.

Category	Subcategory	Desired position					
		Staff (n = 166)		Supervisor (n = 135)		Manager (n = 22)	
		n	(%)	n	(%)	n	(%)
Goals	Specialist	10	[6]	3	[2]	1	[5]
	Generalist	16	[10]	9	[7]	2	[9]
	Establishment and improvement of status and significance of public health dieticians	1	[1]	4	[3]	0	(0)
	Increase and secure public health dieticians	6	[4]	4	[3]	0	(0)
	Establishment and expansion of public health dieticians work	3	[2]	2	[1]	0	(0)
	Assignment of public health dietitians to departments other than health and hygiene-related departments	2	[1]	5	[4]	1	[5]
Health promotion activities	Nutrition education	2	[1]	2	[1]	0	(0)
	Food environment	0	(0)	3	[2]	0	(0)
	Community assessment	4	[2]	3	[2]	0	(0)
	Health emergency management	0	(0)	1	[1]	0	(0)
	Healthy environment	2	[1]	0	(0)	0	(0)
	Public nutrition activities in general	4	[2]	2	[1]	0	(0)
	Public health activities in general	6	[4]	6	[4]	1	[5]
Organizational activities	Improvement of work efficiency	2	[1]	5	[4]	0	(0)
	Human resource development	3	[2]	10	[7]	3	[14]
	Creating a comfortable working environment	3	[2]	3	[2]	2	[9]
Evaluation from others	Evaluation from organization	10	[6]	3	[2]	1	[5]
	Evaluation from residents	4	[2]	1	[1]	2	[9]
	Visualization of achievements	6	[4]	8	[6]	1	[5]
	Appeal achievements to others	5	[3]	2	[1]	1	[5]
Collaboration	Collaborate with dietitians	10	[6]	11	[8]	2	[9]
	Cooperation with stakeholders	7	[4]	8	[6]	1	[5]
	Cooperation and cooperation with residents	3	[2]	4	[3]	0	(0)
	Collaborate with other occupations	13	[8]	16	[12]	2	[9]
Skills to be acquired	Expert knowledge/skills	10	[6]	9	[7]	0	(0)
	Acquisition of qualification	1	[1]	1	[1]	1	[5]
	Ideas that respond to times and flexible idea	8	[5]	7	[5]	1	[5]
	Ability to show business	2	[1]	1	[1]	0	(0)
	Communication skill	4	[2]	3	[2]	0	(0)
	Presentation skill	3	[2]	0	(0)	0	(0)
	Administrative knowledge/skill	6	[4]	8	[6]	1	[5]
	General knowledge	2	[1]	1	[1]	0	(0)
	Planning and formation of project skill	1	[1]	5	[4]	0	(0)
	Negotiation/coordination skill	0	(0)	2	[1]	0	(0)
	Evaluation analysis ability	7	[4]	2	[1]	1	[5]
	Consensus building ability	0	(0)	0	(0)	0	(0)
	Problem analysis ability	3	[2]	5	[4]	1	[5]
Means for improving skills	Workshop participation	6	[4]	8	[6]	3	[14]
	Self-improvement	0	(0)	0	(0)	0	(0)
	Job rotation	6	[4]	0	(0)	1	[5]

Public health dietitians working in municipalities who want to continue their current positions as staff frequently described “public nutrition activities in general,” “public health activities in general” as [health promotion activities], “evaluation from the organization” as [evaluation from others], “collaboration with other occupations” as [collaboration], and “expert knowledge/skill” and “general knowledge” as [skills to be acquired] (Table 4). Those who wanted to be in a supervisory position frequently described “increasing and securing public health dietitians” as [goals], and “public nutrition activities in general” and “general public health activities” as [health promotion activities], “collaboration with dietitians”, “collaboration with stakeholders”, and “collaboration with other occupations” as [collaboration], “expert knowledge/skill”, and “administrative knowledge/skill” as [skill desired to be acquired], and “participation in workshops” as [means for improving skills]. From those whose desired position was a managerial position, “increasing and securing public health dietitians” as [goals], “improvement of working environment” as [organizational activities], “evaluation within the organization” as [evaluation from others], “collaboration with other occupations” as [collaboration], and “expert knowledge/skill, “ideas that respond to times and flexible idea”, and “administrative knowledge/skill” as skills desired to be acquired were often described.

**Table 4:** The vision and skill improvement that public health dietitians working in municipalities want to achieve in 10 years.

Category	Subcategory	Desired position					
		Staff (n = 521)		Supervisor (n = 328)		Manager (n = 182)	
		n	(%)	n	(%)	n	(%)
Goals	Specialist	10	[2]	11	[3]	2	[1]
	Generalist	18	[3]	12	[4]	4	[2]
	Establishment and improvement of status and significance of public health dieticians	13	[2]	8	[2]	5	[3]
	Increase and secure public health dieticians	17	[3]	18	[5]	9	[5]
	Establishment and expansion of public health dieticians work	5	[1]	4	[1]	1	[1]
	Assignment of public health dietitians to departments other than health and hygiene-related departments	4	[1]	2	[1]	4	[2]
Health promotion activities	Nutrition education	23	[4]	10	[3]	7	[4]
	Food environment	4	[1]	5	[2]	3	[2]
	Community assessment	6	[1]	12	[4]	3	[2]
	Health emergency management	3	[1]	6	[2]	3	[2]
	Healthy environment	2	(0)	3	[1]	0	(0)
	Public nutrition activities in general	31	[6]	24	[7]	7	[4]
	Public health activities in general	52	[10]	24	[7]	15	[8]
Organizational activities	Improvement of work efficiency	5	[1]	3	[1]	1	[1]
	Human resource development	8	[2]	9	[3]	6	[3]
	Creating a comfortable working environment	9	[2]	8	[2]	9	[5]
Evaluation from others	Evaluation from organization	31	[6]	12	[4]	15	[8]
	Evaluation from residents	22	[4]	10	[3]	3	[2]
	Visualization of achievements	19	[4]	9	[3]	4	[2]
	Appeal achievements to others	3	[1]	4	[1]	3	[2]
Collaboration	Collaborate with dietitians	20	[4]	18	[5]	7	[4]
	Cooperation with stakeholders	19	[4]	17	[5]	8	[4]
	Cooperation and cooperation with residents	15	[3]	10	[3]	2	[1]
	Collaborate with other occupations	39	[7]	29	[9]	17	[9]
Skills to be acquired	Expert knowledge/skills	44	[8]	21	[6]	12	[7]
	Acquisition of qualification	7	[1]	5	[2]	0	(0)
	Ideas that respond to times and flexible idea	17	[3]	13	[4]	10	[5]
	Ability to show business	8	[2]	1	(0)	1	[1]
	Communication skill	13	[2]	9	[3]	3	[2]
	Presentation skill	5	[1]	0	(0)	1	[1]
	Administrative knowledge/skill	19	[4]	25	[8]	23	[13]
	General knowledge	26	[5]	12	[4]	8	[4]
	Planning and formation of project skill	13	[2]	7	[2]	8	[4]
	Negotiation/coordination skill	3	[1]	5	[2]	1	[1]
	Evaluation analysis ability	6	[1]	6	[2]	3	[2]
	Consensus building ability	0	(0)	0	(0)	0	(0)
	Problem analysis ability	5	[1]	3	[1]	2	[1]
Means for improving skills	Workshop participation	20	[4]	15	[5]	6	[3]
	Self-improvement	19	[4]	7	[2]	3	[2]
	Job rotation	6	[1]	3	[1]	3	[2]

**Fig. 1:**
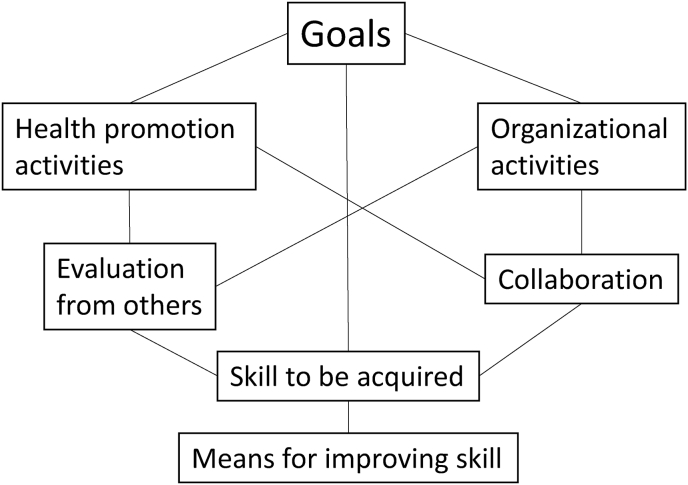
The co-occurrence of these seven categories.

Fig. 1 shows the co-occurrence of these seven categories. When the [goal] of the participants was a “specialist,” [health promotion activities] were often written about “public nutrition,” and the [skills to be acquired] were often “expert knowledge/skill”. On the other hand, when the [goal] of participants was a “generalist”, descriptions about “public health” were often written in the [health promotion activities], and the [skills to be acquired] were often “administrative knowledge/skill”. There was little relationship between the desired positions. It was stated that participants wanted to improve their skills to obtain positive evaluations from others, to obtain positive evaluations from others in their health promotion activities, and to create a work environment where they could receive positive evaluations from others. It was also stated that collaboration was essential for both health promotion and organizational activities and that they wanted to achieve the goal through collaboration. In addition, it was stated that they wanted to increase the number of public health dietitians, improve their positions, and establish the significance of their existence by gaining evaluations from others. It was stated that establishing the task of public health dietitians to work in cooperation with each other by being assigned to health- and sanitation-related departments was necessary.

## 4 Discussion

Schein indicated the vertical, horizontal, and central directions of movement of the carrier stage of the external career [12]. External career stages may or may not reflect a person's internal career [9]. Although the position desired in this study corresponds to the vertical direction of the career stage, the results did not reflect the inner career.

This study examined the characteristics of public health dietitians' visions for skill improvement for the next 10 years, according to their desired positions. This analysis enabled us to classify them into seven categories. Since Japanese public health dietitians work in circumstances where there are generally a small number of the same profession per organization [11], we discussed six categories that are strongly related to the work environment of Japanese public health dietitians.

### 4.1 Goals

The “specialist” and “generalist” indicated by the individuals’ [goal] of position type correspond to the technical/functional competence and general managerial competence indicated by Schein et al. [9]. for career anchors, respectively. The career ladder of public health nurses [13] and the career advancement of the Japan Dietetic Association [14] also indicate the direction of career advancement by dividing into generalists in practice and specialists in specialized fields.

Dietitians play an important social role and are required to have a high level of expertise; however, they do not have sufficient working conditions or social status to meet those requirements in Japan. The Japan Dietetic Association [15] and the Japan Association of Dietitian Training Institution [16] aim to improve the working status of dietitians, which needs improvement to achieve their vision [17]. The status of hospital dietitians is not equal to that of other medical workers in Japan [18]. Although there is progress in the sharing of occupational fields among medical and welfare workers [19], the work of public health dietitians has not yet been established.

### 4.2 Organizational activities

Approval of superiors in the workplace can help to maintain job satisfaction and pride in the profession [20]. Currently, the tasks of public health dietitians are not well understood by other occupations within the organization, which may decrease the amount of approval they receive from superiors [21]. While there are generally multiple public health nurses at organizations, typically public health dietitians work alone. Therefore, public health dietitians must carry out their work efficiently to complete their large workloads, which takes up time that could be used for human resource development. There are also difficulties in the preceptor system that public health nurses use.

### 4.3 Evaluation from others

There have been some reports on the need to increase the social awareness of public health dietitians. The Ministry of Health, Labour, and Welfare reported that the role of dietitians should be widely known to the public and that further promotion of nutrition care activities in the community should be promoted [22]. This report implies that dietitians do not have high social demand and that their evaluation rates are low. Although dietitians themselves understand the importance of their duties and the high level of expertise required to do their work, it is possible that they are not evaluated appropriately because they are not understood by others. As the Japanese public health dietitians tend to be in a one-person workplace, it is difficult to assert themselves because they depend on other professions [23].

### 4.4 Collaboration

As dietitians often work independently, they are not good at coordinating with other occupations despite a general need to do so [24]. Public health dietitians should work at an organization where multiple public health dietitians work so their careers can progress. If this isn't possible, collaboration with other professionals is necessary.

### 4.5 Skills to be acquired

Katz advocates that managers need to have technical, human and conceptual skills [25]. In the subcategories of [skills to be acquired] that were extracted in this study, “expert knowledge/skill”, “qualification acquisition”, “ideas that respond to times and flexible ideas” correspond to technical skills, “skill for appealing achievements”, “communication skill”, and “presentation skill” correspond to human skills and “administrative knowledge/skill”, “general knowledge”, “skill for planning and formation of projects”, “negotiation and coordination skill”, “management skill”, “evaluation skill”, “consensus building skill”, and “problem analysis ability” correspond to conceptual skill. In a one-person workplace, one professional is representative of the entire organization. Therefore, because people see them as specialized occupations, it is difficult for them to understand what they do, which may change based on the needs of society at that time, such as the COVID-19 pandemic.

### 4.6 Means for improving skills

When there are multiple professionals in the workplace, it is possible to teach and learn from one another. However, there are no such opportunities in one-person workplaces. Moreover, in a one-person workplace, it is difficult to participate in workshops during working hours, and self-improvement is often deprioritized. According to Nakanishi et al.^17)^, to realise the future vision of dietitians, it is necessary to improve the system to make it easier to participate in workshops and academic conferences, assign dietitians, increase the number of dietitians, provide an environment and place where they can learn and receive lifelong education. To improve skills, it is necessary not only to provide educational opportunities but also to improve the workplace environment.

This study has two limitations. First, it was not possible to calculate an accurate response rate despite obtaining information from the Japan Dietetic Association and the Japanese Association of Public Health Center. From the statistics offered by the Ministry of Health, Labor, and Welfare, our survey recovery rate from public health dietitians working in cities and special wards with public health centers, and municipalities was less than half. Therefore, the results of this study do not necessarily reflect the experiences of all public health dietitians in Japan. Second, in this study, we analyzed the target position type, but there was a positive correlation between the target position type, work history and current position of health promotion in the community. Because of this, the results of this study may reflect the fact that the target position type is limited to work history and the current position of health promotion in the community.

## 5 Conclusion

We conducted a survey of the vision for skill improvement over the next 10 years of Japanese public health dietitians working in prefectures, cities, municipalities and special wards with public health centers. This vision can be broadly divided into visions of nutrition specialists and visions of public health generalists. The percentage did not show much relationship with the target position type. The skills they wanted to improve differed depending on their vision, but evaluations of the project and cooperation from others were described regardless of what they wanted to achieve. This study suggests that human resource development programs for public health dietitians need to incorporate the skills they want to improve, pathways for different target position types (nutrition specialist or public health generalist) and tools to work collaboratively with others so they can receive positive evaluations.

## Ethical approval

Procedures for this study were followed in accordance with the ethical standards of the Helsinki Declaration and were approved by the Ethical committee of Osaka City University (Institutional Review Board protocol 20–27, approval date: October 14, 2020).

## Funding

This study was supported by Health and Labour Sciences Research Grants of the 10.13039/501100003478Ministry of Health, Labour and Welfare, Japan (Comprehensive Research on Life-Style Related Diseases including Cardiovascular Diseases and Diabetes Mellitus.

## Declaration of competing interest

There are no conflicts of interest to declare.
